# Construction and validation of a meropenem-induced liver injury risk prediction model: a multicenter case-control study

**DOI:** 10.3389/fphar.2025.1542554

**Published:** 2025-05-09

**Authors:** Yan He, Hongqin Ke, Jianyong Zhu, Xin Yuan, Hongliang Li, Wenwen Wu, Shuman Yang, Huibin Yu

**Affiliations:** ^1^ Department of Pharmacy, Renmin Hospital, Hubei University of Medicine, Shiyan, Hubei, China; ^2^ School of Pharmaceutical Sciences, Hubei University of Medicine, Shiyan, Hubei, China; ^3^ Department of Pharmacy, Taihe Hospital, Hubei University of Medicine, Shiyan, Hubei, China; ^4^ Department of Respiratory Medicine, Renmin Hospital, Hubei University of Medicine, Shiyan, Hubei, China; ^5^ Hubei Key Laboratory of Wudang Local Chinese Medicine Research, Hubei University of Medicine, Shiyan, Hubei, China; ^6^ Department of Preventive Medicine, School of Public Health, Hubei University of Medicine, Shiyan, Hubei, China; ^7^ Department of Endocrinology, The First Affiliated Hospital of Jinzhou Medical University, Jinzhou, Liaoning, China; ^8^ Department of Epidemiology and Biostatistics, School of Public Health, Jilin University, Changchun, Jilin, China

**Keywords:** drug-induced liver injury, meropenem, risk factor, prediction model, drug safety, adverse drug reaction

## Abstract

**Objective:**

To construct and validate a risk prediction model for patients with meropenem-induced liver injury (MiLI).

**Methods:**

A retrospective case-control study was conducted to collect data on inpatients treated with meropenem at Shiyan People’s Hospital, Hubei, China from January 2018 to December 2022; this study served as the model construction dataset. Univariate analysis and multiple logistic regression analysis were employed to identify the related factors for MiLI, and a nomogram risk prediction model for MiLI was constructed. The recognition ability and prediction accuracy of the model were evaluated using the receiver operating characteristic (ROC) and calibration curves. The clinical efficacy was assessed via the decision curve analysis (DCA). The internal validation was performed using the bootstrap method, and external validation was conducted based on an external dataset from Shiyan Taihe Hospital between October 2021 and December 2023.

**Results:**

A total of 1,625 individuals were included in the model construction dataset, of which 62 occurred MiLI. The external validation dataset included 1,032 cases, with 74 patients developing liver injury. Six variables were independent factors for MiLI and included in the final prediction model: being male (OR = 2.080, 95% CI: 1.050–4.123, *P* = 0.036), ICU admission (OR = 8.207, 95% CI: 4.094–16.453, *P* < 0.001), gallbladder disease (OR = 8.240, 95% CI: 3.605–18.832, *P* < 0.001), baseline ALP (OR = 1.012, 95% CI: 1.004–1.019, *P* = 0.004), GGT (OR = 1.010, 95% CI: 1.005–1.015, *P* < 0.001), and PLT (OR = 0.997, 95% CI: 0.994–0.999, *P* = 0.020). The *c-*statistic value for internal validation of the prediction model was 0.821; the sensitivity and specificity were 0.997 and 0.924, respectively. The *c-*statistic value of the prediction model in the model construction dataset was 0.837 (95% CI, 0.789–0.885), while in the external validation dataset was 0.851 (95% CI, 0.802–0.901). The *P*-values of the calibration curve in the two datasets were 0.935 and 0.084, respectively.

**Conclusion:**

Being male, ICU admission, gallbladder disease, higher levels of baseline ALP and GGT, and lower levels of baseline PLT were the risk factors for MiLI. The nomogram model built based on these factors demonstrated favorable performance in discrimination, calibration, clinical applicability, and internal-external validation. The nomogram model can assist clinicians in early identification of high-risk patients receiving meropenem, predicting the risk of MiLI, and ensuring safe medication practices.

## 1 Introduction

Meropenem is a broad-spectrum carbapenem antibiotic used to treat gram-positive and gram-negative bacteria infections. It has efficacy in treating the moderate to severe bacterial infections, mixed infections, and infections caused by multidrug-resistant bacteria (Gobezie et al., 2024). With the increasing clinical use of meropenem, there has been a growing incidence of liver injury reported in association with its administration (Cheung et al., 2021; Tattersall et al., 2018; Zubarev et al., 2020). Drug-induced liver injury (DILI) is primarily caused by biological agents, chemicals, and other factors that cause damage to liver (Mili et al., 2023). An epidemiological study showed that the annual incidence of DILI among hospitalized patients in China is 23.80 per 100,000, which is higher than the rate reported in Western countries (Shen et al., 2019). DILI is one of the most common and serious adverse drug reactions encountered in clinical practice (Tuohutaerbieke et al., 2021; Zeng et al., 2022). In severe cases, DILI can directly lead to liver failure and even death.

Until now, there are only several case reports on meropenem-induced liver injury (MiLI) (Cheung et al., 2021; Tattersall et al., 2018; Zubarev et al., 2020). These case report studies primarily focused on investigating the types and characteristics of liver injury associated with meropenem. There is a notable deficiency in research concerning the influencing factors and predictive models for MiLI. Consequently, this study conducted a retrospective case-control analysis of hospitalized patients treated with meropenem. We aimed to identify the risk factors for MiLI and construct a nomogram risk prediction model. This model is intended to assist clinical staffs in early identification of MiLI risk, facilitate prevention measures in clinical practice, and enhance meropenem safety use.

## 2 Materials and methods

### 2.1 Basic information of subjects

The data were collected from hospitalized patients treated with meropenem at the Shiyan People’s Hospital between January 2018 and December 2022. For external validation, we extracted data from hospitalized patients using meropenem at the Taihe Hospital in Shiyan City from October 2021 to December 2023. The same inclusion and exclusion criteria were applied to both datasets. Inclusion criteria were shown as follows: ① Hospitalized patients receiving meropenem; ② Age ≥18 years old; ③ A complete liver biochemical examination was conducted before and after medication; ④ No history of DILI prior to medication. Exclusion criteria included: ① other diseases that may cause abnormal liver function, such as fatty liver, liver cancer, viral hepatitis, ischemic or autoimmune hepatitis, alcoholic liver disease, and malignant tumors (Zeng et al., 2022); ② death; and ③ incomplete clinical data.

### 2.2 Definition of MiLI and the grouping of patients

According to the criteria for DILI outlined in the 2023 edition of the Chinese Guidelines for the Diagnosis and Treatment of DILI, the liver biochemical indices must meet at least one of the following requirements (Li et al., 2022a): ① ALT ≥5×ULN; ② ALP ≥2×ULN; or ③ ALT ≥3×ULN and TBIL ≥2×ULN. The Roussel Uclaf Causality Assessment Method (RUCAM) Scale was employed to evaluate the causal relationship between liver injury and meropenem (Rodríguez et al., 2022). The patients in the case group met one of the biochemical criteria for DILI, and had the RUCAM score is ≥3 points. Patients without liver injury after meropenem use served as the controls.

### 2.3 Data collection

As suggested by a previous research (Li et al., 2022b), the following factors included in this study: ① general information such as sex, age, underlying diseases, medical history, concomitant medications, and duration of meropenem use; ② baseline laboratory test values, including ALT, AST, ALP, TBIL, GGT, ALB, CREA, PLT, WBC, HGB, PT, PCT and INR, biochemical assay methods followed those described in the references (Shang et al., 2019). Baseline data of laboratory tests were the most recent test values prior to the use of meropenem (Ji et al., 2023). All MiLI cases were evaluated and reviewed by two senior or above clinical pharmacists.

### 2.4 Statistical analysis

The SPSS (version 26.0) and R 4.4.1 for Windows were used for statistical analysis. For quantitative data, the normality of the data was assessed using the Shapiro-Wilk test prior to the parametric tests. Data that conformed to a normal distribution were expressed as mean ± standard deviation, and comparisons between the case and control groups were conducted using the independent two-sample *t*-test. For data that did not follow a normal distribution, data were presented as median and interquartile ranges, and the Mann-Whitney *U* test was employed for comparisons between the case and control groups. Categorical data were expressed as n (%) and the comparisons between the case and control groups were analyzed using the chi-squared (*χ* ^*2*^) test.

Variables with *P* < 0.05 in univariate analysis were included in the initial multivariable model, and variables with statistical significance in the multivariate logistic regression analysis were included in the final prediction model. Multivariable logistic regression was performed to identify independent risk factors for MiLI; odds ratios (ORs) and 95% confidence intervals (CIs) were also estimated. Two-sided *P* < 0.05 were considered to be statistically significant.

The risk prediction model for MiLI was established based on the independent risk factors and presented in the form of a nomogram. The discriminative ability of logistic regression model was assessed based on the area under the receiver operating characteristic (ROC) curve. Model calibration was evaluated using the Hosmer-Lemeshow statistics. The clinical effectiveness was evaluated by the decision curve analysis (DCA). We used the Bootstrap method to calculate the *c*-statistics of the model. External validation dataset also used to evaluate the predictive accuracy of the model.

## 3 Results

### 3.1 Basic information of the research object

In Shiyan People’s Hospital, 4,456 hospitalized patients aged ≥18 years treated with meropenem were initially screened. After excluding 2,831 cases (1,042 with other liver injury-causing diseases, 366 deaths, and 1,423 with incomplete clinical data), 1,625 cases (62 cases and 1,563 controls) were included in the model construction dataset. Univariate and multivariate logistic analyses were performed on this dataset to construct a predictive model for MiLI. Internal validation was conducted via bootstrap resampling using the same dataset.

**FIGURE 1:**
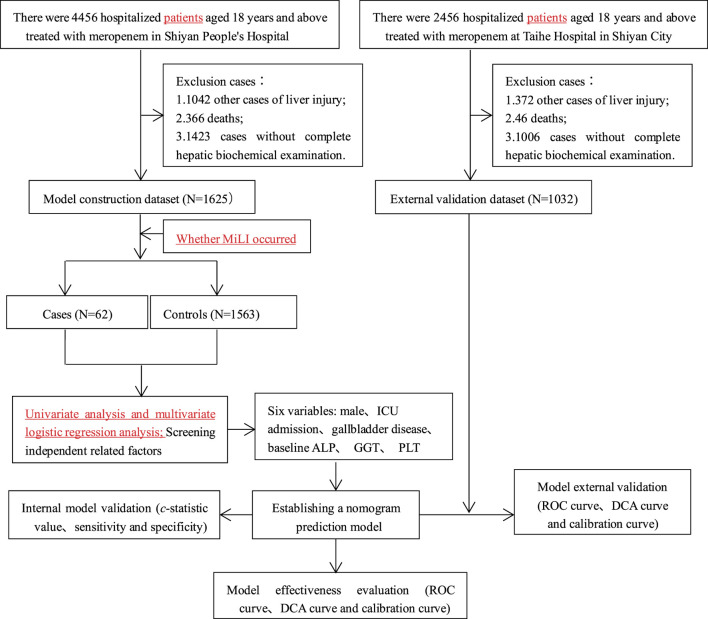
Flow chart for prediction model establishment and validation.

At the Taihe Hospital in Shiyan City, 2,456 hospitalized patients aged ≥18 years received meropenem treatment. We excluded 1,424 cases (372 had liver injury from other diseases, 46 died, and 1,006 had incomplete clinical data). The external validation dataset comprised 1,032 cases (74 cases and 958 controls), which were used to evaluate the model’s predictive performance. The research flow chart is illustrated in Figure 1.

### 3.2 Univariate analysis and multivariate logistic regression analysis

Univariate analysis was conducted to compare the cases and the controls within the model construction dataset (Table 1). Significant differences between the cases and the controls were observed for sex, length of stay, hypoproteinemia, shock, ICU admission, sepsis or septicemia, liver disease, gallbladder disease, cardiovascular disease, as well as the laboratory baseline ALT, ALP, GGT, AST, CREA, PLT, and PCT (*P* < 0.05). The variable coding was shown in Supplementary Table S1.

**TABLE 1:** Univariate analysis of MiLI related variables in the model construction dataset.

Variable	Controls N = 1,563	Cases N = 62	*χ* ^*2*^ */Z/t*	*P*
Sex [n (%)]			4.775	0.029
Yes	1,026 (65.64%)	49 (79.03%)		
No	537 (34.36%)	13 (20.97%)		
Allergy history [n (%)]			0.025	0.875
Yes	135 (8.64%)	5 (8.06%)		
No	1,428 (91.36%)	57 (91.94%)		
Smoking history [n (%)]				
Yes	574 (36.72%)	22 (35.48%)	0.040	0.842
No	989 (63.27%)	40 (64.52%)		
Alcohol history [n (%)]			1.340	0.247
Yes	472 (30.20%)	23 (37.10%)		
No	1,091 (69.80%)	39 (62.90%)		
Diabetes [n (%)]			0.825	0.364
Yes	282 (18.04%)	14 (22.58%)		
No	1,281 (81.96%)	48 (77.42%)		
Hypertension [n (%)]			1.237	0.266
Yes	670 (42.87%)	31 (50.00%)		
No	893 (57.13%)	31 (50.00%)		
Hypoproteinemia [n (%)]			9.572	0.002
Yes	556 (35.57%)	34 (54.84%)		
No	1,007 (64.43%)	28 (45.16%)		
Shock [n (%)]			5.668	0.017
Yes	250 (15.99%)	17 (27.42%)		
No	1,313 (84.01%)	45 (72.58%)		
ICU admission [n (%)]			40.740	<0.001
Yes	624 (39.92%)	50 (80.65%)		
No	939 (60.08%)	12 (19.35%)		
Sepsis or septicemia [n (%)]			4.137	0.042
Yes	313 (20.03%)	19 (30.65%)		
No	1,250 (79.97%)	43 (69.35%)		
Liver disease [n (%)]			6.804	0.009
Yes	218 (13.95%)	16 (25.81%)		
No	1,345 (86.05%)	46 (74.19%)		
Gallbladder disease [n (%)]			41.714	<0.001
Yes	43 (2.75%)	11 (17.74%)		
No	1,520 (97.25%)	51 (82.26%)		
Kidney diseases [n (%)]			1.164	0.281
Yes	408 (26.10%)	20 (32.26%)		
No	1,155 (73.90%)	42 (67.74%)		
Cardiovascular disease [n (%)]			5.414	0.020
Yes	1,011 (64.68%)	49 (79.03%)		
No	552 (35.32%)	13 (20.97%)		
Nervous disease [n (%)]				
Yes	729 (46.64%)	35 (56.45%)	2.304	0.129
No	834 (53.36%)	27 (43.55%)		
Respiratory disease [n (%)]			0.007	0.934
Yes	1,242 (79.46%)	49 (79.03%)		
No	321 (20.54%)	13 (20.97%)		
Other antimicrobial agents [n (%)]			2.147	0.143
Yes	1,431 (91.55%)	60 (96.77%)		
No	132 (8.45%)	2 (3.23%)		
Lipid-lowering drug [n (%)]			0.570	0.450
Yes	557 (35.64%)	25 (40.32%)		
No	1,006 (64.36%)	37 (59.68%)		
Antipyretic and analgesic [n (%)]				
Yes	280 (17.91%)	15 (24.19%)	1.583	0.208
No	1,283 (82.09%)	47 (75.81%)		
Antiepileptic drug [n (%)]			0.366	0.545
Yes	187 (11.96%)	9 (14.52)		
No	1,376 (88.04%)	53 (85.48%)		
Antihypertensive drug [n (%)]			1.081	0.299
Yes	702 (44.91%)	32 (51.61%)		
No	861 (55.09%)	30 (48.39%)		
Age (years)	67.56 (54.75,76.79)	67.17 (56.72,77.11)	−0.504	0.614
Length of stay (days)	17.00 (11.71,26.15)	24.00 (14.75,37.25)	−3.602	<0.001
ALT (U/L)	16.75 (11.00,27.92)	24.00 (15.50,48.25)	−3.704	<0.001
TBIL (μmol/L)	11.90 (8.30,17.50)	11.90 (7.83,17.73)	−0.009	0.993
ALP(U/L)	56.00 (43.00,73.00)	64.00 (44.50,119.25)	−2.391	0.017
GGT (U/L)	24.00 (15.00,42.23)	45.50 (19.75,81.75)	−3.670	<0.001
AST (U/L)	21.00 (15.00,35.00)	33.00 (19.00,53.50)	−4.249	<0.001
ALB (g/L)	33.60 (30.10,37.00)	32.65 (28.80,36.50)	−1.430	0.153
TP (g/L)	61.80 (55.90,67.60)	59.60 (53.30,67.55)	−1.037	0.300
TBA (μmol/L)	4.00 (2.00,7.80)	5.90 (2.55,8.48)	−1.759	0.079
CREA (μmol/L)	80.85 (64.75,110.40)	93.85 (74.63,122.63)	−2.591	0.010
PT(s)	13.60 (12.30,15.30)	14.10 (12.48,15.85)	−0.976	0.329
INR	1.12 (1.02,1.25)	1.16 (1.05,1.30)	−1.284	0.199
TT(s)	15.30 (14.00,16.90)	15.10 (13.88,17.25)	−0.410	0.682
WBC(×10^9^/L)	9.41 (6.35,13.22)	10.68 (7.70,13.41)	−1.282	0.200
LYM(×10^9^/L)	0.82 (0.54,1.23)	0.86 (0.57,1.23)	−0.518	0.604
EOS(×10^9^/L)	0.01 (0.00,0.08)	0.01 (0.00,0.06)	−0.168	0.867
HGB (g/L)	111.47 ± 24.76	105.53 ± 22.84	1.856	0.064
PLT (×10^9^/L)	180.00 (125.00,245.00)	145.50 (95.50,203.75)	−2.533	0.011
PCT (ng/mL)	0.28 (0.16,1.28)	0.52 (0.21,2.00)	−2.789	0.005
hs-CRP (mg/L)	62.95 (21.16,94.08)	69.88 (28.56,113.95)	−1.085	0.278

Note: ① Liver disease excluded HAV, HBV, HCV, HEV, autoimmune hepatitis, fatty liver, alcoholic liver, liver malignancy and other diseases, and the presence of liver abscess, liver cyst, liver nodules and other mild basic liver disease; ② Gallbladder disease excluded PBC, PSC, pancreatic bile duct malignancy and other diseases, existing in the past cholecystitis, gallstones, post-cholecystectomy and other diseases; ③ Cardiovascular disease included the clinical diagnosis of coronary heart disease, heart failure, myocardial infarction, angina pectoris and others.

A total of 16 variables with significant differences in univariate analysis were included in the multivariate logistic regression analysis. Variables such as hypoproteinemia and shock which showed insignificant *P* were excluded from the multivariate logistic regression model. As shown in Table 2, six variables with statistical significance were finally included in the multivariate logistic regression model: being male [OR 2.080 (1.050–4.123), *P* = 0.036], ICU admission [OR 8.207 (4.094–16.453), *P* < 0.001], gallbladder disease [OR 8.240 (3.605–18.832), *P* < 0.001], baseline ALP [OR 1.012 (1.004–1.019), *P* = 0.004], GGT [OR 1.010 (1.005–1.015), *P* < 0.001], and PLT [OR 0.997 (0.994–0.999), *P* = 0.020].

**TABLE 2:** Multiple logistic regression analysis of MiLI related factors in the model construction dataset.

Variable	*β*	OR	95% *CI*	*P*
Sex (Male)	0.732	2.080	1.050–4.123	0.036
ICU admission	2.105	8.207	4.094–16.453	<0.001
Gallbladder disease	2.109	8.240	3.605–18.832	<0.001
ALP	0.011	1.012	1.004–1.019	0.004
GGT	0.010	1.010	1.005–1.015	<0.001
PLT	−0.003	0.997	0.994–0.999	0.020

Note: *β*, regression coefficient; *OR*, odds ratio; *CI*, confidence interval.

**FIGURE 2:**
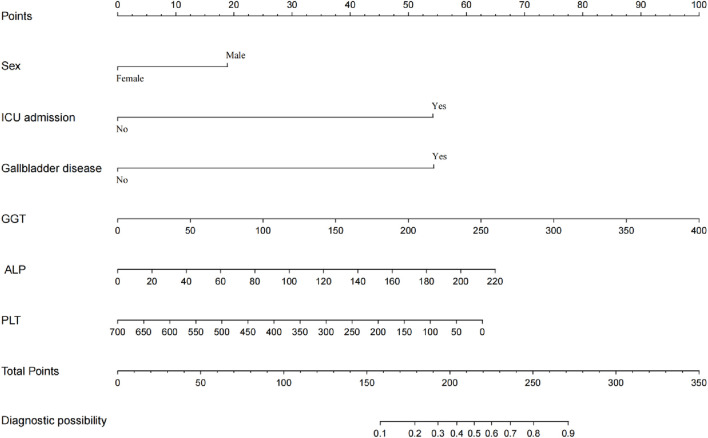
Nomogram for predicting MiLI.

The final prediction model was constructed based on the variables showed significant relationship with MiLI. As shown in Figure 2, the prediction model was presented in the form of a nomogram. Each measurement variable is aligned vertically with the top reference score line to derive the corresponding score. The total score is calculated by summing the scores of all variables. The probability of MiLI occurrence can be determined by matching the total score vertically with the risk line.

### 3.3 Validation of the prediction model

**FIGURE 3:**
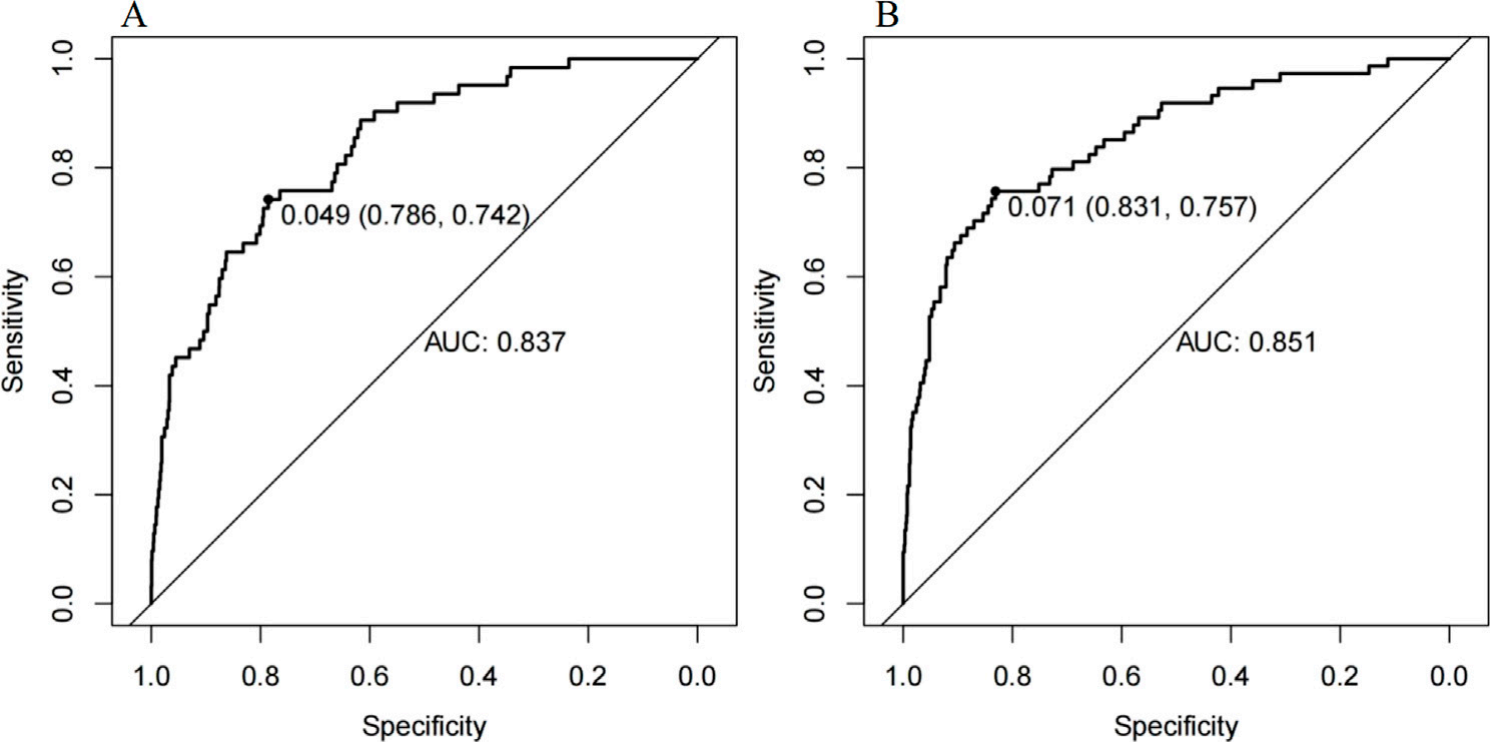
The ROC curves for the MiLI prediction model in model construction dataset **(A)** and external validation dataset **(B)**.

**FIGURE 4:**
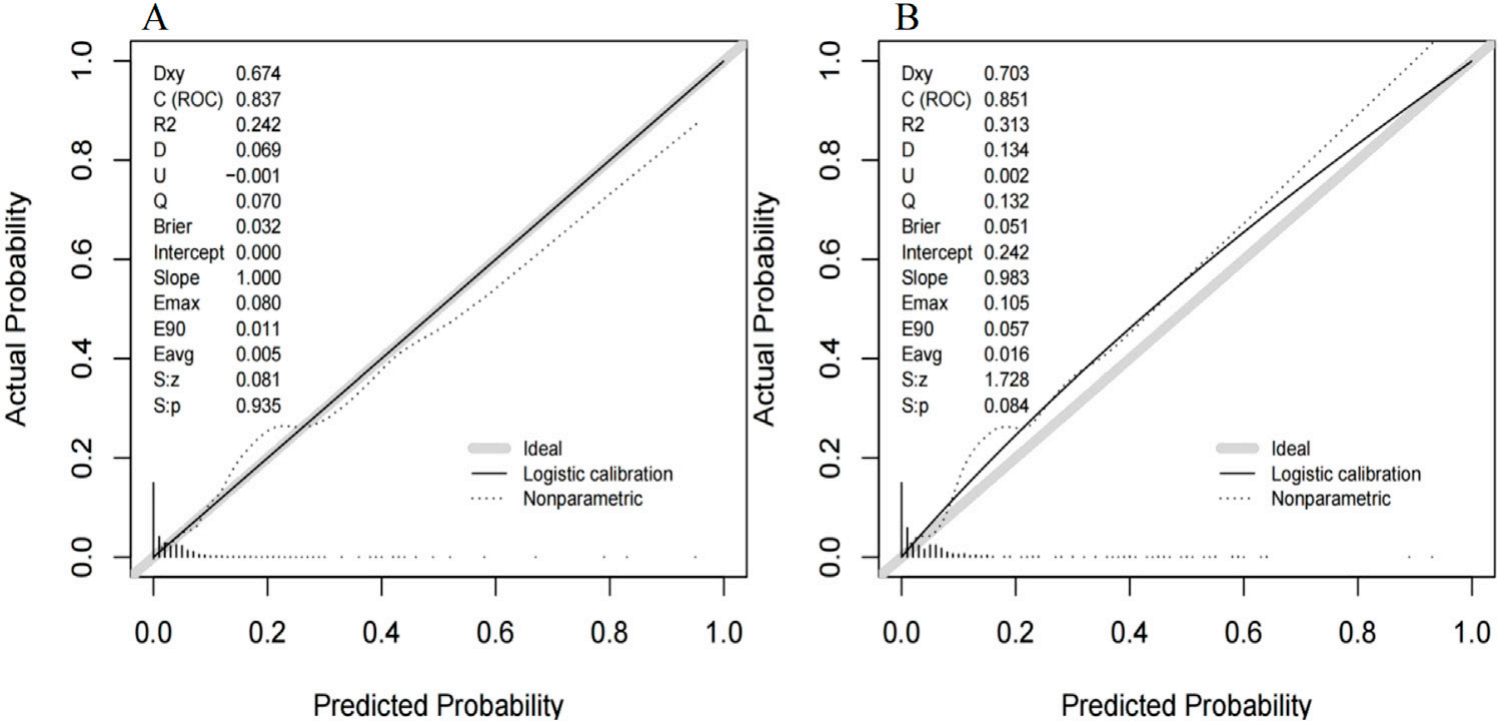
The calibration curve for the MiLI prediction model in model construction dataset **(A)** and external validation dataset **(B)**. Note: The calibration curve is the black solid line in the figure, the ideal curve is the grey solid line in the figure, and the nonparametric curve is the dashed line in the figure.

**FIGURE 5:**
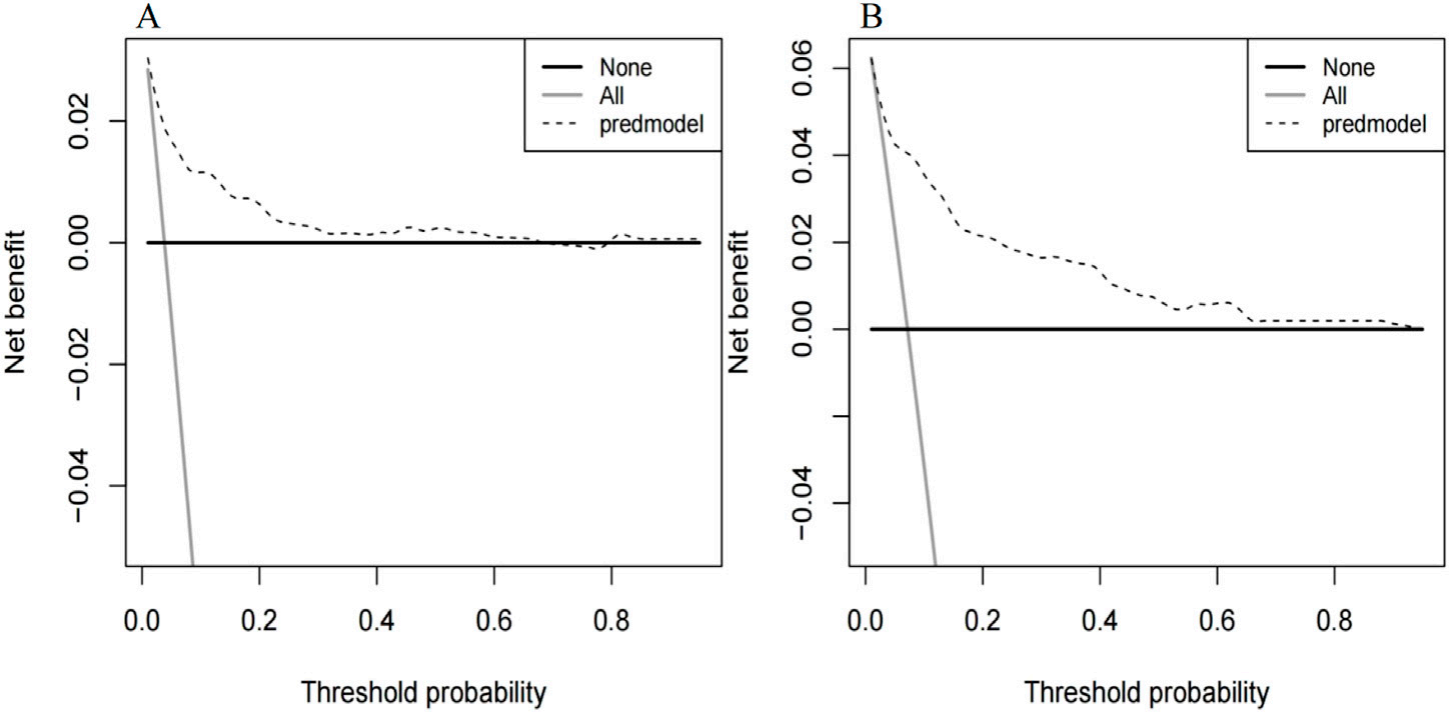
Decision curves for the MiLI prediction model in model construction dataset **(A)** and external validation dataset **(B)**. Note: The DCA curve is the dashed line in the figure. The two extreme reference curves, the None line and the All line, are the grey solid line and the black solid line, respectively.

The ROC curve of the model construction dataset is shown in Figure 3A, the prediction model had a *c*-statistic of 0.837, sensitivity of 0.742 (95% CI: 0.633∼0.851), and specificity of 0.786 (95% CI: 0.765∼0.806). The calibration curve of the model construction dataset is presented in Figure 4A, the Hosmer-Lemeshow goodness-of-fit test indicated a *P*-value of 0.935 for the prediction model. The DCA curve of the model construction dataset is shown in Figure 5A, the DCA curve was higher than the other two extreme curves between 1% and 70%. The Bootstrap method was used to extract 1,000 times for internal verification, and the *c-*statistic value was 0.821, sensitivity 0.997, and specificity 0.924. To ensure the reliability of our prediction model, model built based on construction dataset was also externally validated. The ROC curve of the external validation dataset is shown in Figure 3B, with a *c*-statistic of 0.851, sensitivity of 0.757, and specificity of 0.831. The calibration curve of the external validation dataset is presented in Figure 4B, and the Hosmer-Lemeshow goodness-of-fit test indicated a *P*-value of 0.084. The DCA curve of the external validation dataset is shown in Figure 5B, the DCA curve was higher than the other two extreme curves between 1% and 92%.

## 4 Discussion

Case reports of MiLI have been reported (Cheung et al., 2021), but there is a lack of research on the risk factors and predictive models for MiLI. This study retrospectively collected data on hospitalized patients using meropenem at two centers. The analysis showed that being male, ICU admission, gallbladder disease, higher levels of baseline ALP and GGT, and lower levels of baseline PLT were the risk factors for MiLI. Based on these factors, a prediction model for MiLI was established and presented in the form of a nomogram. The effectiveness evaluation, internal and external verification showed that the nomogram had a good predictive accuracy.

Akimoto et al. (2021) extracted data from adult patients across two databases and found that meropenem was associated with a higher risk of DILI. According to the DILI criteria, a total of 1,625 hospitalized patients treated with meropenem at the Shiyan People’s Hospital from January 2018 to December 2022 were included in the model construction dataset. Among these patients, there were 62 cases of liver injury and 1,563 cases without liver injury, resulting in an incidence of MiLI of 3.82%. This finding is consistent with the incidence of 0.1%–5% in a previous study (Wu et al., 2019).

In this study, six factors were significant factors for the incident MiLI. The impact of sex on DILI remains a topic of debate. Bonkovsky et al. (2014) reported that women exhibit greater susceptibility to DILI than men when exposed to certain medications, such as minocycline and furantoin. However, other studies suggested that the risk of DILI is higher in males than in females (Zhong et al., 2021; Lin et al., 2024). Shiraishi et al. (2024) demonstrated that male is an independent risk factor for liver injury associated with carbapenem drugs; this indicates that sex should be considered for medication use. In this study, multivariate logistic regression analysis revealed that male is an independent factor for MiLI. Among patients treated with meropenem, the risk of liver damage was found to be 2.08 times higher in men than in women. The higher risk of DILI in males may be associated with sex hormone-mediated differences in hepatic drug-metabolizing enzymes (Yang et al., 2021). Disease severity was closely associated with the incidence of DILI. Yu et al. showed that admission to the ICU was a significant risk factor for the development of DILI in patients treated with tigecycline (Yu et al., 2022); such finding is in line with the results in our study. Patients in the ICU are more susceptible to ischemia-hypoxia reperfusion injury, immune-mediated injury, and systemic inflammatory responses, all of which may increase the risk of DILI (Horvatits et al., 2019). Studies have demonstrated that concomitant biliary disease elevates the risk of DILI (Eder et al., 2014); this is also consistent with our study. Cholecystectomy may induce or exacerbate insulin resistance in susceptible individuals and alter bile acid metabolism, bile accumulates in the bile ducts, raising the ductal pressure which compresses the intrahepatic bile ducts, causing poor intrahepatic bile excretion (Víctor et al., 2017); this may lead to cholesteric liver injury. Following the administration of meropenem, a portion of the drug or its metabolites must be excreted via the biliary tract. Bile duct injuries can easily cause inflammation, which may directly spread to the liver tissue; this causes *in vivo* cholestasis and subsequent liver cell damage (Zhang et al., 2023). It has been reported that MiLI predominantly manifests as cholestatic liver injury (Cheung et al., 2021; Tattersall et al., 2018). Cholestasis *in vivo* is closely associated with higher levels of baseline ALP and GGT. Lv et al. (2023) identified baseline ALP level as a significant factor for tacrolimus-induced DILI. Everhart and Wright (2013) noted that GGT serves as an independent predictor of virological response and clinical outcomes in patients with liver disease. In our study, the higher levels of baseline ALP and GGT were risk factors for MiLI. Elevated ALP levels are associated with oxidative stress in the body (Dai et al., 2021). When oxidative stress manifests in the liver, there is an elevation in reactive oxygen species (ROS) levels and a diminished capacity of the body’s antioxidant defense mechanisms; this results in hepatocyte apoptosis and tissue damage (Villanueva-Paz et al., 2021). Additionally, GGT serves as a marker of oxidative stress, and elevated GGT levels cause the metabolism of glutathione (GSH), and production of ROS and free radicals (Everhart and Wright, 2013; Dominici et al., 2005). GSH has a protective effect on the liver, and ROS and free radical production have been shown to have exacerbate liver damage (Press et al., 2023). Consequently, elevated baseline GGT levels may increase the risk of DILI. In addition, the analysis presented in this study indicates that the lower levels of baseline PLT are a significant factor for MiLI. This finding is consistent with the results reported by Zhang and Liu (2024) and Wang et al. (2022). Furthermore, PLT levels are inversely associated with the severity of liver disease (Gao et al., 2015). Lower levels of PLT are independent predictors for DILI. Liver damage leads to decreased PLT levels in the body (Han et al., 2020). Concurrently, the inflammatory response caused by liver injury causes damages to endothelial cells and platelets (Christophe and Jean-Louis, 2018); this further reduce the level of PLT in the body.

Studies have indicated that broad-spectrum antibiotics such as tigecycline (Yu et al., 2022), amoxicillin-clavulanate, and piperacillin (Pedraza et al., 2021) may also cause DILI. However, univariate analysis in this study showed insignificant results. The reasons for this discrepancy may be related to the differences in patient demographics, drug metabolism, environmental factors, and other variables (Li et al., 2022b).

The differentiation, calibration, and clinical applicability of the prediction model were evaluated using the ROC curve, calibration curve, and DCA curve (Xie et al., 2024). The *c*-statistic value of the ROC curve was 0.837, indicating that the model demonstrated a good differentiation. The calibration curve of the prediction model was close to the ideal model represented by the diagonal dotted line; this shows that the predicted results of the model were consistent with the actual observed values. The calibration degree of the model was good. The DCA curve was higher than the other two extreme curves within the range of 1%–70%. Patients in this range experienced some clinical net benefit. The model underwent internal validation using the bootstrap method. The *c*-statistic value of 0.821 suggests that the model had a good stability. Furthermore, the ROC curve, calibration curve, and DCA curve for the external validation dataset were plotted using the same model. The *c*-statistic value for the external validation dataset was 0.851, reflecting a good accuracy of the prediction model. The calibration curve of the external verification dataset was also close to the ideal model represented by the diagonal dotted line; such finding suggests that prediction model had a good consistency. The DCA curve of the external validation dataset was higher than the two extreme curves within the range of 1%–92%, suggesting the potential clinical application value in this range. The above results showed that the prediction effect of the model is good among different hospitals, and it has universal applicability. This nomogram model can be converted into a dynamic web-based version for clinical application (Wang et al., 2024), such as establishing an individualized prediction platform on the hospital’s official website using this nomogram (Wu et al., 2022), facilitating the convenient calculation of MiLI probability in hospitalized patients and prompting clinical staff to implement measures that ensure the safety of meropenem use.

This study has some limitations. First, it was a retrospective analysis; Second, the data were collected from two hospitals located in the same geographic area, which may limit the representativeness of the sample; Third, we excluded individuals with missing clinical data, this may introduce selection bias. Potential population selection bias cannot be excluded and excluding cases with incomplete data may underestimate the true risk of MiLI. Additionally, the study lacks prospective validation, and future studies should be conducted through multicenter prospective studies to further validate our findings.

## 5 Conclusion

This study retrospectively collected data from hospitalized patients treated with meropenem across two centers. Being male, ICU admission, gallbladder disease, higher levels of baseline ALP and GGT, and lower levels of PLT were the risk factors for MiLI. Based on these factors, a risk prediction nomogram model for MiLI was constructed, and validated internally and externally. In conclusion, the nomogram prediction model can facilitate the early identification of MiLI. Such finding maybe useful for the preventions of MiLI.

## Data Availability

The raw data supporting the conclusions of this article will be made available by the authors, without undue reservation.

## Abbreviations

DILI, Drug-induced liver injury; MiLI, Meropenem-induced liver injury; ROC, Receiver operating characteristic curve; DCA, Decision curve analysis; CI, Confidence interval; RUCAM, Roussel Uclaf Causality Assessment Method; ALT, Alanine aminotransferase; ALP, Alkaline phosphatase; TBIL, Total bilirubin; AST, Aspartate aminotransferase; GGT, Gamma-glutamyl transpeptidase; ALB, Albumin; CREA, Creatinine; PLT, Platelet; WBC, White blood cell; HGB, Hemoglobin; PT, Prothrombin time; PCT, Procalcitonin; INR, International normalized ratio; HAV, Hepatitis A virus; HBV, Hepatitis B Virus; HCV, Hepatitis C virus; HEV, Hepatitis E virus; PBC, Primary biliary cholangitis; PSC, Primary sclerosing cholangitis; GSH, Glutathione; ROS, Reactive oxygen species.
