# Supplementary material for: Construction and validation of a meropenem-induced liver injury risk prediction model: a multicenter case-control study
Source: Front Pharmacol. 2025 May 9;16:1542554. doi: 10.3389/fphar.2025.1542554 (PMC12098429; doi:10.3389/fphar.2025.1542554)
Supplement: Supplementary file 1 [file Table1.docx]

Supplement TABLE S1 Independent variable coding in the multivariate logistic regression analysis

| Independent Variable | Code |
| --- | --- |
| Dependent variable | 1=Cases; 0=Controls |
| Sex | 1=Male; 0=Female |
| Hypoproteinemia | 1=Yes; 0=No |
| Shock | 1=Yes; 0=No |
| ICU admission | 1=Yes; 0=No |
| Sepsis or septicemia | 1=Yes; 0=No |
| Liver disease | 1=Yes; 0=No |
| Gallbladder disease | 1=Yes; 0=No |
| Cardiovascular disease | 1=Yes; 0=No |
